# Inference of Disease-Related Molecular Logic from Systems-Based Microarray Analysis

**DOI:** 10.1371/journal.pcbi.0020068

**Published:** 2006-06-16

**Authors:** Vinay Varadan, Dimitris Anastassiou

**Affiliations:** Department of Electrical Engineering and Center for Computational Biology and Bioinformatics (C2B2), Columbia University, New York, New York, United States of America; University of California Berkeley, United States of America

## Abstract

Computational analysis of gene expression data from microarrays has been useful for medical diagnosis and prognosis. The ability to analyze such data at the level of biological modules, rather than individual genes, has been recognized as important for improving our understanding of disease-related pathways. It has proved difficult, however, to infer pathways from microarray data by deriving modules of multiple synergistically interrelated genes, rather than individual genes. Here we propose a systems-based approach called Entropy Minimization and Boolean Parsimony (EMBP) that identifies, directly from gene expression data, modules of genes that are jointly associated with disease. Furthermore, the technique provides insight into the underlying biomolecular logic by inferring a logic function connecting the joint expression levels in a gene module with the outcome of disease. Coupled with biological knowledge, this information can be useful for identifying disease-related pathways, suggesting potential therapeutic approaches for interfering with the functions of such pathways. We present an example providing such gene modules associated with prostate cancer from publicly available gene expression data, and we successfully validate the results on additional independently derived data. Our results indicate a link between prostate cancer and cellular damage from oxidative stress combined with inhibition of apoptotic mechanisms normally triggered by such damage.

## Synopsis

Diseases such as cancer are often associated with malfunctioning pathways involving several genes. Identifying modules of such genes and how the genes in each module interact with each other is helpful toward understanding the nature of these diseases. Here the authors provide a novel computational method for discovering such modules of genes merely from two sets of gene expression data, one from healthy tissues and one from tissues suffering from a particular disease. The method is based on the concept of identifying sets of genes whose joint expression state predicts the presence or absence of a particular disease with minimum uncertainty. Once such gene sets have been identified, we can then further use the microarray data to determine the “logic” that connects the genes' individual expression states related to the outcome of the disease. In turn, this logic may give us valuable insight into the nature of the pathways and how we may target some elements of these pathways for therapeutic purposes. The authors apply this methodology in a particular example and conclude that prostate cancer is often associated with cellular damage from oxidative stress combined with the inhibition of the apoptotic mechanisms normally activated by such damage.

## Introduction

The expression levels of thousands of genes, measured simultaneously using DNA microarrays, provide information useful for medical diagnosis and prognosis [1,2]. However, their ability to provide significant help towards devising therapeutic approaches has not yet been demonstrated. This failure can be partly attributed to the fact that traditional gene selection techniques typically end up producing a “list of genes” that are correlated with disease, without providing insights into the mutual interrelationships of these genes.

Gene selection techniques from microarray analysis are often based on individual gene ranking depending on a numerical score measuring the correlation of each gene with particular disease types. The expression levels of the highest-ranked genes tend to be either consistently higher in the presence of disease and lower in the absence of disease, or vice versa. Such genes usually have the property that their joint expression levels corresponding to diseased tissues and the joint expression levels corresponding to healthy tissues can be cleanly separated into two distinct clusters. These techniques are therefore convenient and powerful for classification purposes between disease and health, or between different disease types, but they are incompatible with a systems biology viewpoint, because they do not identify systems of synergistically interacting genes, whose joint expression state predicts disease. Rather, microarray clustering techniques tend to produce clusters of co-regulated genes.

Sophisticated machine learning classification approaches, in which a hypersurface on a high-dimensional space serves as a classification boundary separating the gene expression points into classes of tissues, have also been successfully used. Certain nonlinear transformations are typically used to define the shape of the hypersurface, but the performance of the algorithms is limited by the ability to identify and use the optimum such transformation. Furthermore, the set of selected genes is typically not combined with an easily interpretable interrelationship among its members, which could otherwise provide biological insight about the combined role of these genes.

To address such problems, several efforts have recently been made to analyze expression data at the level of biological modules, rather than individual genes [3–9]. However, it has proved difficult, so far, to infer modules of multiple synergistically interrelated genes directly from microarray data.

In this paper, we present a systems-based approach (Entropy Minimization and Boolean Parsimony [EMBP]) that identifies modules of genes jointly associated with disease from gene expression data. The technique also produces a simple logic function connecting the combined expression levels in each gene module with the presence of disease. Roughly speaking, the goals of EMBP analysis are, first, to identify the smallest module of genes whose joint expression levels can predict the presence of disease with high accuracy, and, second, to identify the simplest logic function connecting these genes to achieve this prediction. We applied EMBP analysis on a prostate cancer dataset [10], and validated the resulting gene modules and logic functions on a different dataset [11].

## Results

### Binarization of Gene Expression Data

We first binarize microarray expression data into two levels. Although the EMBP methodology can be generalized to account for multiple expression levels, binarization of expression data simplifies the presentation of the concepts in this paper and provides simple logical functions connecting the genes within the found modules.

Rather than independently binarizing each gene's expression level, which would be more appropriate for an individual gene ranking approach, we chose to use single thresholds for all genes. This approach is consistent with the fact that we seek to find global interrelationships among genes and that the microarray data have already been normalized across all tissues and all genes. Therefore, a choice of high threshold will identify the genes that are “strongly” expressed, while a choice of a low threshold will identify the genes that expressed even “weakly.” We performed EMBP analysis across several thresholds and we focused on the threshold choices that provided best performance, as described in the following sections.

### Entropy Minimization

Following binarization, each gene is assumed to be either expressed or not expressed in a particular tissue, and we also assume that there are two types of tissues, either healthy ones or tissues suffering from a particular disease. The latter assumption can also be generalized to include more than two types of tissues, or modified to be used for classification among several disease types.

Thus, given *M* genes and *K* tissues, an *M × K* binary “expression matrix” *E* is defined so that *E(i,j)* is 1 if gene *i* is expressed in tissue *j,* and 0 otherwise. Furthermore, a *K*-vector *c* is defined so that *c(j)* is 1 if tissue *j* is diseased and 0 if it is healthy. For each gene module of size *n,* there are 2*^n^* possible gene expression states, and for each state *S* we can count the number *N*_0_(*S*) of times that the state appears in a healthy tissue, and the number *N*_1_(*S*) of times that it appears in a diseased tissue. We can then create a table with 2*^n^* rows corresponding to the gene expression states, which we refer to as the “state-count table” in which each row contains the two counts *N*_0_ and *N*_1_ for the corresponding state. Table 1 shows two examples of such state-count tables for *n =* 4.

**Table 1:**
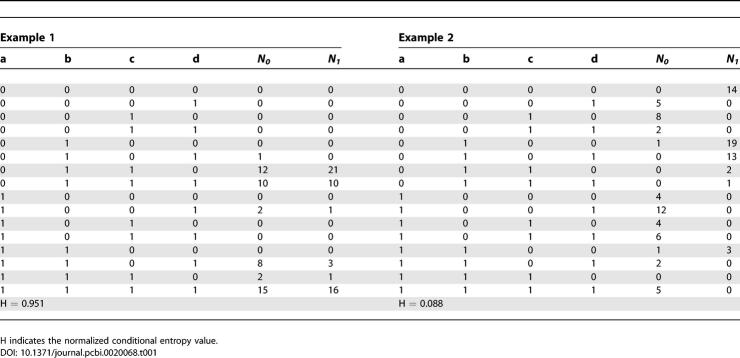
Two Examples of State-Count Tables and the Corresponding Normalized Conditional Entropies

We first address the following problem:

Given a number *n,* identify the set of *n* genes whose combined expression levels predict the presence or absence of disease with minimum uncertainty.

We refer to this problem as the “entropy minimization” problem, because we quantify the uncertainty with the information-theoretic measure known as conditional entropy [12] after creating a probabilistic model in which probabilities are equal to relative frequencies derived from the counts *N*_0_(*S*) and *N*_1_(*S*), so that the presence of disease and the gene expression states are random variables.

In the following, we define the conditional entropy and explain in what sense it measures uncertainty. Given a discrete random variable satisfying a probability distribution {*p_i_*}, the entropy 
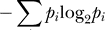
 is, in rough terms, the “average length of the shortest description” of the value of the variable [12]. More formally, if we have a sequence of independently drawn symbols, all of which obey an identical probability distribution, then it is a result proven by Shannon [13] that the entropy of that probability distribution measures the minimum average number of bits per symbol required to describe their values. Similarly, the conditional entropy of a random variable, given another variable, is defined as the expected value of the entropies of the conditional distributions, averaged over the conditioning random variable [12], and it measures the average length of the shortest description of the value of one random variable given the value of another. For our purposes, assume that the binary random variable *C* describes the presence or absence of a particular disease, and that *S* is the random variable (binary n-vector) describing the expression state of a particular gene set of size *n*. The quantity that we wish to minimize is the conditional entropy, *H*(*C*|*S*), of *C* given *S*.

Specifically, using the counts *N*_0_ and *N*_1_, we define

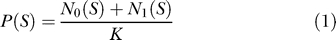
as the probability of encountering expression state *S* in a tissue chosen at random, and

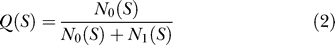
as the probability of disease in a tissue, given that its expression state is *S,* where Equation 2 is applied for the states that have been encountered at least once.

If we know the expression state *S* for a particular tissue, then the uncertainty of determining whether or not disease exists in that tissue is measured by the entropy *H*(*Q*(*S*)), where the function *H* is defined by

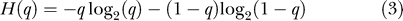
Note that the function *H*(*q*) becomes close to 0 for values of *q* that are close to either 0 or 1, and takes a maximum value of 1 for *q* = 0.5, which is consistent with its interpretation as uncertainty. The average overall uncertainty of determining whether or not disease is present is then measured by the conditional entropy of the presence of disease given the expression state for the gene set:

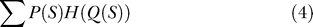
where the summation is over all 2*^n^* states *S* with *P*(*S*) > 0. Again, this formula is consistent with the intuitive interpretation of uncertainty, because it becomes small whenever the probabilities *Q*(*S*) become close to either 0 or 1 for the more frequently encountered states.

Finally, to ensure that the range of possible values for the conditional entropy extends from 0 to 1, we normalize by dividing by *H*(*Q*_null_), the entropy corresponding to the probability of disease in a randomly chosen tissue. (In the case of the prostate data that we use, this probability is equal to 52/102). For simplicity, in the sequel we will often refer to the normalized conditional entropy as just “entropy.”

The conditional entropy, as defined above, depends on the counts *N*_0_ and *N*_1_ for the 2*^n^* states. Its interpretation as a measure of uncertainty is illustrated in the example of Table 1, which contains two state-count tables that were created using the binarized expression matrix for the 102 prostate tissues used in this paper, and a threshold of 15. The state-count table on the left corresponds to a choice of four genes a, b, c, and d, selected at random. The resulting value of the normalized conditional entropy of 0.951 is typical for random choices of gene sets. On the other hand, the state-count table on the right corresponds to the gene set for which we found the minimum normalized conditional entropy of 0.088, consisting of genes a: *COL4A6,* b: *CYP1B1,* c: *SERPINB5,* and d: *GSTP1,* to be discussed later in this paper. In this latter gene set choice, as shown in Table 1, the reduced entropy is manifested by the fact that the statistics are skewed for nearly all states. For example, all 13 tissues corresponding to state 0101 are cancerous, and all 12 issues corresponding to state 1001 are healthy.

The entropy minimization problem consists of identifying the gene set with the minimum conditional entropy, as defined above, among all subsets of size *n* of the full set of *M* genes. The number of these subsets is equal to 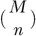 and becomes large for *n* ≥ 3, making the exhaustive search method impractical. As explained in the Materials and Methods section below, however, this problem can easily be addressed using heuristic search optimization methods.

If the conditional entropy for a particular gene set is found to be exactly 0, this implies that the joint expression levels of the members of that gene module determine the existence of disease with absolute certainty under the assumption of the probabilistic model derived from the relative frequencies. This happens whenever, for all 2*^n^* states in the corresponding state-count table, at least one of the counts *N*_0_ and *N*_1_ is 0. In our experiments, we have found that when this occurs, a large number of states are only encountered once or twice. We cannot make any reliable association of disease based on these rarely encountered states, and including them in our model will result in “overfitting,” so we treat them as noise and ignore them, in favor of the states which predominantly correspond to disease. Our definition for such states is that they have been encountered at least three times and that the number of corresponding diseased tissues is at least four times larger than the count of corresponding healthy tissues, i.e., *N*_1_ ≥ 3 and *N*_1_ ≥ 4*N*_0_. Therefore, whenever we find the entropy for a gene set to be exactly 0, we decrease the size of the gene set by 1, and select the minimum-entropy gene set of that size. Thus, the output of the EMBP analysis contains a gene module for which the conditional entropy is close, but not equal, to 0.

Calculating the conditional entropy of a particular set of genes of length *n* involves evaluating the counts *N*_0_(*S*) and *N*_1_(*S*) for each of the 2*^n^* states. However, the number of states for which these counts are non-zero cannot be larger than the number *K* of tissues. Thus the computational cost of calculating the entropy of a gene set remains bounded regardless of the size of the gene set.

### Boolean Parsimony

Once a gene module has been identified, and the expression states for that module that are predominantly associated with disease have been determined as described above, we then address the following problem: Given the gene expression states associated with disease, find the simplest logical rule that connects the expression levels in the gene module with the presence of disease.

We refer to this problem as the “Boolean parsimony” problem, because the logical rule will be identified by the “most parsimonious Boolean function.” Our definition for this logic function is one containing the operators AND, OR and NOT, which minimizes a “cost,” defined as the total number of logic variables appearing in the expression. In Boolean algebra [14], each logic variable can take the value of either 0 (false) or 1 (true), the operator AND corresponds to multiplication, and the operator OR corresponds to addition. We use the symbol of prime (′) following the logic variable to designate the operator NOT. For example, ab+a′b′+ab′ means (a AND b) OR [(NOT a) AND (NOT b)] OR [a AND (NOT b)] and the “cost” (as defined above) of this Boolean function is 6, because each of the variables a and b appears three times. This Boolean expression happens to be logically equivalent to a+b′, meaning: a OR (NOT b). The latter expression is more parsimonious than the former, because its “cost” is equal to 2, as each of the letters a and b appears once.

The reason for the need of Boolean parsimony is that the biological role of each gene becomes more immediately clear if the Boolean expression contains the corresponding logic variable either once or only a few times. We selected the above definition of Boolean parsimony because the logic functions AND, OR, and NOT often have straightforward potential biological interpretations.

The problem is easily solved manually when the size of the gene set is less than 5, as in the examples of this paper, using Karnaugh map logic design methodology [15] (Figure 1). Otherwise, Boolean minimization programs such as Espresso [16] can be used. Most of them retain the “sum of products” structure of the Boolean expression, but further minimization is desirable and possible using heuristic algorithms [17].

**Figure 1:**
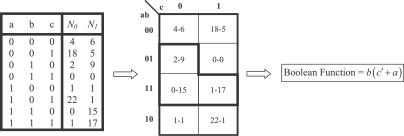
Example of Estimating the Boolean Function for a Gene Module The state-count table for a set of three genes is used to create the 4 × 2 table shown on the right, known as a “Karnaugh map.” The binary coordinates in the rows and columns of Karnaugh maps are arranged according to the “Gray code” (consecutive coordinates differ by one bit only) for easier derivation of the logical function. Each entry of the Karnaugh map corresponds to one of the states and the numbers shown are equal to the counts *N*_0_ and *N*_1_ for that state. In this example, there are three entries (010, 110, 111) identified as predominantly associated with a particular disease. States 010 and 110 can be jointly described by the function bc′. Similarly, states 110 and 111 can be jointly described by the function ba. The overall Boolean function describing the area highlighted by bold lines in the Karnaugh map is bc′+ba = b(c′+a).

The computational cost of Boolean Parsimony is insignificant compared to that of Entropy Minimization. For example, Espresso [16] takes less than a minute on a standard Pentium III processor running at 3 GHz for Boolean functions involving many tens of variables. The BDS algorithm [17] used to find the most parsimonious Boolean function also takes less than a minute for functions having many tens of Boolean variables.

### Prostate Cancer EMBP Analysis

We used two different prostate cancer datasets. The first prostate cancer microarray expression data [10] contain gene expression profiles for 102 prostate tissues, of which 52 were cancerous and 50 were healthy and is available in the public domain from http://www-genome.wi.mit.edu/MPR/prostate. The gene expression profiles in scaled average difference units were produced using HG-U95A Affymetrix microarrays with probes for 12,600 genes (Affymetrix, Santa Clara, California, United States). This dataset will henceforth be referred to as the “EMBP dataset,” because we used it to apply EMBP analysis. A second independently derived dataset, also containing scaled average difference units referring to 34 tissue samples of which 25 were cancerous and nine were healthy was also obtained from the public domain [11] (http://www.gnf.org/cancer/prostate) and used for validating the gene modules and logic functions estimated over the EMBP dataset. This latter dataset will be referred to as the “validation dataset.”

#### Gene module and Boolean function identification using the EMBP dataset.

Each threshold choice for binarizing the continuous-valued data of the EMBP dataset will produce potentially different results. The “optimum” threshold can be defined and evaluated as the one that yields the minimum overall entropy. In our case, we found that this would be equal to 60. However, EMBP analysis is not meant to produce a unique gene set as its output. Rather, it can reveal a rich content of information from the microarray data by using different threshold choices. The combined information resulting from several such gene sets can further help provide insight into related pathways.

We used several thresholds ranging from −30 to 225 in steps of 15 and estimated the minimum entropy gene modules for each of them. For each threshold value we considered gene modules of size *n* = 1, 2, 3, and 4. For *n* = 4, we found that the entropy values occasionally went down to precisely 0 due to overfitting. On the other hand, we found several gene sets of size 3 with entropy values less than 0.20, which is low—note that H(0.97) = 0.20, meaning that if the conditional entropy is 0.20, then, on the average, each state is associated with either cancer or health with probability 97%. Therefore, we selected *n* = 3 to be the number of genes included in these gene modules. Our results of are shown in Figure 2A. The thresholds for which the minimum entropy values were below 0.20 for *n* = 3 are 30, 45, 60, 75, and 90. The minimum entropy gene modules for these thresholds along with their entropies are listed in Table 2. In the following, we use the official gene symbols, and Table 3 contains a legend with the corresponding accession numbers, aliases, and brief gene descriptions.

**Figure 2:**
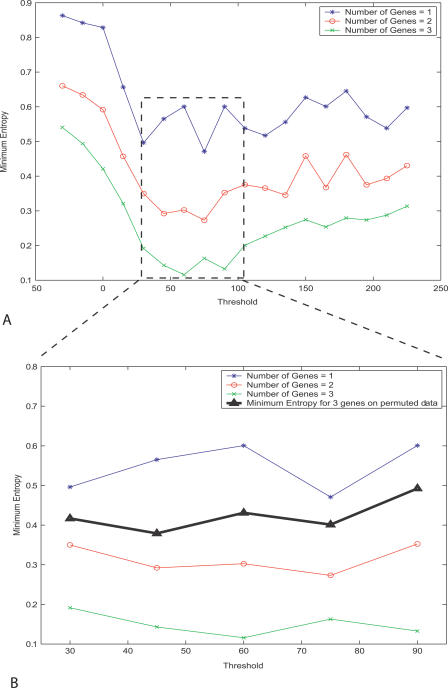
Minimum Entropy across Different Thresholds (A) Estimates of the minimum entropy values for gene modules of size *n* = 1, 2, and 3 across various thresholds. (B) Minimum entropy values for subset of thresholds (colored lines) along with the estimated means of the entropies over randomly permuted data for *n* = 3 (black line).

**Table 2:**
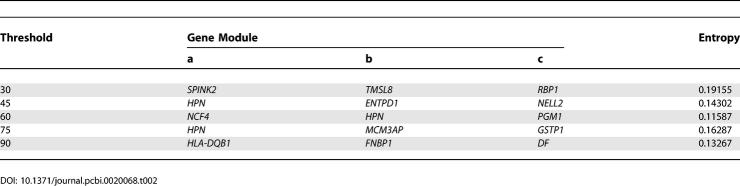
List of the Minimum Entropy Gene Modules for Different Thresholds

**Table 3:**
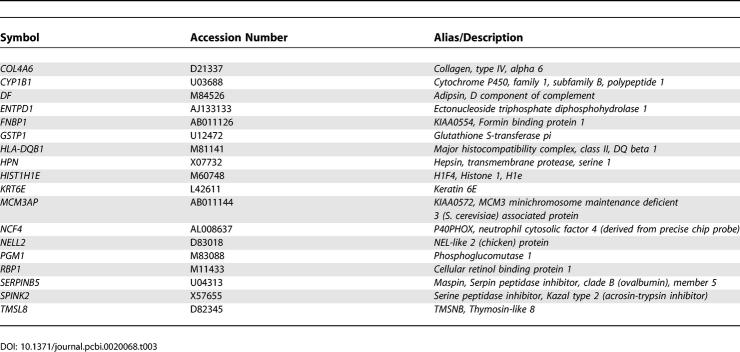
List of Genes That Were Included in the Estimated Gene Modules along with Their Accession Numbers and Gene Description

To evaluate the significance of these minimum entropy values, we performed entropy minimization over ten random permutations of the tissue class labels. In other words, while keeping the number of healthy and cancerous tissues constant to 50 and 52, respectively, we randomly assigned healthy (0) and cancerous (1) labels to the individual tissue profiles. The entropy minimization algorithm was performed on the randomly permuted data, and the average minimum entropies for *n* = 3 were estimated for the thresholds 30, 45, 60, 75, and 90 for the same expression matrix of the EMBP dataset. The estimated averages of the entropies are shown in the heavy black line in Figure 2B. Notably, the entropy values for the randomly permuted data for *n* = 3 are much higher than those estimated on the actual dataset, and even significantly higher than the entropy values of the actual data with *n* = 2, indicating that the gene modules identified by entropy minimization on the actual data have real biological meaning, rather than being due to chance. To further emphasize this fact, we performed entropy minimization over 40 random permutations of the tissue class labels using the E matrix for threshold 60, chosen because it yields the lowest overall entropy. We observed that the 40 entropy values derived from the permuted data consistently fit a normal distribution using any of the chi-square, Lillie and Geary tests [18]. The mean and standard deviation of the entropy values were 0.4557 and 0.0433, respectively. Using these statistics, we estimated the *p*-value of the minimum entropy on the actual data, defined as the probability of the minimum entropy being not more than 0.11587 purely by chance to be extremely small, equal to 2 × 10^−15^.

We then estimated the most parsimonious Boolean functions for these five gene modules, which were selected because the corresponding thresholds are close to each other and spread around the “optimum” one, equal to 60, as mentioned above. Figure 3 contains the Karnaugh maps from which the functions were derived, together with the corresponding Boolean functions and their accuracy if these simple functions are used for classification on the EMBP dataset. For convenience, these Boolean functions are also formulated in words in Figure 3, where “presence” and “absence” of a gene refer, for simplicity, to the presence or absence of mRNA from the gene. Furthermore, we found that gene *ENTPD1* in Figure 3B can be replaced by gene *HIST1H1E,* and that gene *NCF4* in Figure 3C can be replaced by gene *KRT6E*. In both cases, these substitutions yield identical results.

**Figure 3:**
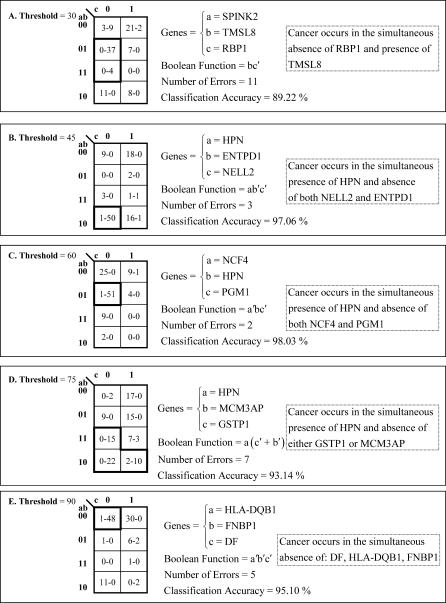
Karnaugh Maps Leading to Boolean Functions across Different Thresholds Shown are the Karnaugh maps for the minimum entropy three-gene modules for five choices of binarization threshold, the parsimonious Boolean functions derived from the Karnaugh maps, and the classification accuracy of these Boolean functions over the EMBP dataset. Furthermore, we found that gene *ENTPD1* in Figure 3B can be replaced by gene *HIST1H1E* and that gene *NCF4* in Figure 3C can be replaced by gene *KRT6E*. In both cases, these substitutions yield identical results, but are not shown in the figure, for simplicity.

The genes mentioned in Figure 3 should not be seen as individual “prostate cancer-related genes,” which, in traditional approaches, are found to be either consistently overexpressed or consistently underexpressed in prostate cancer. Instead, each of the identified genes should be seen as a member of a synergistic gene module, as evidenced by the formulation of the corresponding Boolean function. To further clarify the fundamental difference between the two approaches, we mention the following “notable facts” for each of the five identified gene modules, derived from simple observation of the counts in each Karnaugh map, each of which could provide hints for its biological explanation:

(1) Absence of *RBP1,* if accompanied by either presence of *TMSL8* or absence of *SPINK2,* is associated with cancer in 50 out of 53 such tissues. However, in the simultaneous absence of *TMSL8* and presence of *SPINK2,* absence of *RBP1* is not associated with cancer. On the contrary all 11 such tissues are healthy.

(2) Presence of *NELL2* is associated with health in 37 out of 39 such tissues, even if *HPN* (normally associated with cancer) is present. Simultaneous presence of *HPN* and *NELL2* is associated with health in 17 out 19 such tissues.

(3) Presence of *NCF4* is associated with health in all 11 such tissues, even if *HPN* (normally associated with cancer) is present: Simultaneous presence of *HPN* and *NCF4* is associated with health in all nine such tissues. The same formulation is true if *NCF4* is replaced by *KRT6E*.

(4) If either *HPN* is present or *MCM3AP* is absent, then the absence of *GSTP1* is associated with cancer, as all such 39 tissues are cancerous. However, if *HPN* is absent and *MCM3AP* is present, then the absence of *GSTP1* is not associated with cancer, as all such nine tissues are healthy.

(5) In the absence of *HLA-DQB1,* absence of *DF* is associated with cancer in 48 tissues out of 50, and presence of *DF* is associated with health in 36 tissues out of 38. However, in the presence of *HLA-DQB1,* absence of *DF* is instead associated with health, as all 11 such tissues are healthy.

#### Validation of the gene modules and Boolean functions using the validation dataset.

Although the classification performance of the Boolean functions from EMBP analysis is high over the dataset upon which the results were derived (Figure 3), it is important to validate these results over previously unseen gene expression profiles. We tested the five gene modules of Figure 3 using their corresponding Boolean functions on the “validation dataset” [11]. For that task, we needed to binarize the expression levels of the validation dataset.

Because of the simplicity of the Boolean functions shown in Figure 3, which are applied to only three genes, *any* choice of threshold for this task yielding good classification results would be remarkable, as a moment's thought would be convincing that a “random” Boolean function cannot have good classification results regardless of the threshold choice. We chose, nevertheless, to find a single transformation formula mapping the EMBP dataset thresholds to the validation dataset thresholds. This is not a straightforward task, because of the lack of standardization in the conditions involved when measurements are made in different laboratories. For example, in our case, the median intensity of the validation dataset was close to ten times that of the EMBP dataset.

We used a simple transformation of the form *y* = *ax* + *b* where *x* represents thresholds over the EMBP dataset and *y* represents thresholds over the validation dataset. To estimate the coefficients *a* and *b,* we averaged the gene expression values over all tissues for the 12,600 genes common to both datasets. We thus obtained two vectors *x* and *y* of length 12,600, whose elements were the mean gene expression levels across all tissues belonging to the EMBP and validation datasets, respectively. Using these two vectors, we calculated the least-squares estimate for the coefficients *a* and *b*. The mean value and the 95% confidence bound for the two coefficients were found to be: *a* = 8.25 ± 0.088 and *b* = 92.12 ± 9.06. We transformed the thresholds using several values of *a* and *b* within the 95% confidence bounds, and selected the values yielding the highest found classification performance, which were *a* = 8.338 and *b* = 92.12. Table 4 summarizes the results for each of the five Boolean functions outlined in Figure 3 over the validation dataset.

**Table 4:**
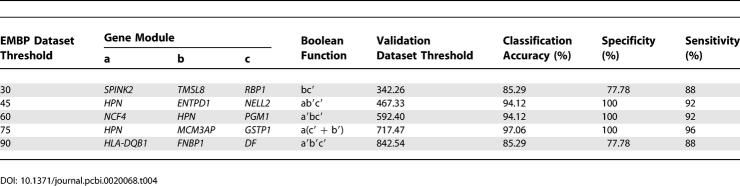
Classification Accuracy of EMBP Analysis Results over the Validation Dataset

Remarkably, the classification accuracy of the simple three-gene (two-gene in one case) Boolean functions in the validation dataset were consistently high, exceeding 90% in most cases, indicating that EMBP analysis accurately extracted universally valid prostate cancer–related features. By comparison, the validation results of the *k*-nearest neighbor-based classification in [10], from which we extracted our input data, using the same training and testing data [11], had classification accuracy of 77% for four-gene models and 86% on 16-gene models, while even our worst accuracy across different thresholds for three-gene classification was 85.29% (see Table 4).

As mentioned earlier, because of overfitting, the classification accuracy on the validation data would not increase had we used four-gene modules for the threshold choices in Table 4. Indeed, the resulting Boolean functions were the same as the Boolean functions for *n* = 3, since the Boolean Parsimony procedure found that the fourth gene in the modules was irrelevant. To illustrate the concept of overfitting, we also evaluated the classification performance of the four-gene modules on the validation datasets by skipping the Boolean Parsimony procedure, instead using the individual states of the four-gene modules as predictors of cancer. In other words, we predicted each state to be cancerous or benign based on the “majority count” calculated from the values of *N*_0_ and *N*_1_ in the training dataset. The classification performance of the four-gene modules across thresholds 30, 45, 60, 75, and 90 was found to be 85.29%, 94.11%, 91.17%, 82.35%, and 79.41% respectively. In other words, the performance of the four-gene modules either worsens or remains the same compared to the three-gene EMBP modules in Table 4. Thus the Boolean Parsimony procedure addresses the problem of overfitting by its very nature.

### Biological Interpretation of EMBP Analysis Results

The genes in the modules resulting from EMBP analysis are not co-regulated, because, if they were, then each of them alone would provide much of the information that all of them provide; therefore a different gene would be a more appropriate partner, as it would provide complementary information. Nevertheless, these genes are typically related by a shared common “theme” in which they are playing synergistic roles. For example, two genes may appear because they are both required for the activation of a particular cancer-causing pathway. Of course, the cause-and-effect relationship connecting disease and the presence of particular genes in a gene module is not clear from the results of quantitative analysis alone, and the Boolean functions can only be seen as approximations when they are based on a relatively small set of input data, as in our case.

Coupled with additional biological knowledge, however, the results of EMBP analysis can help infer disease-related pathways, which, in turn can help develop therapeutic interventions. This methodology uses the clues provided by the results to create speculative assumptions involving additional genes. Assuming that each gene module has a “story” to say, we can then attempt to combine all these “stories” into an integrated scenario combining many genes. In this section, we provide two examples of this methodology.

We first focus on the three-gene module with the lowest overall conditional entropy (0.1159) (Figure 3C), consisting of genes *HPN, NCF4,* and *PGM1*. Hepsin (HPN) is a serine protease that is overexpressed in most prostate cancers [19]. Recent evidence indicates [20,21] that hepsin converts single-chain pro-hepatocyte growth factor into biologically active two-chain hepatocyte growth factor. The hepatocyte growth factor (HGF) is a ligand for Met, a known proto-oncogene receptor tyrosine kinase, suggesting [20,21] that this functional link between hepsin and the HGF/Met pathway may be related to tumor progression. Furthermore, HGF protects cell against oxidative stress–induced apoptosis [22,23]. These results suggest that hepsin may promote tumor progression by inhibiting the apoptotic mechanisms that are normally activated in cells after they become cancerous as a result of damage caused by oxidative stress.

Interestingly, both of the other members of the module *(NCF4* and *PGM1)* have also been related to oxidative stress, strengthening the above speculative interpretation. Phosphoglucomutase is inhibited under oxidative stress [24]. The absence of *PGM1* (as in the Boolean function of Figure 3C) could therefore result from oxidative stress. On the other hand, *NCF4,* also known as *P40PHOX,* is known to down-regulate [25,26], under some conditions, the NADPH oxidase, a phagocyte enzyme system that creates a superoxide-producing “oxidative burst” in response to invasive microorganisms. In this case, local oxidative stress would result from the reduced levels of *P40PHOX* activity.

Taken together, the above observations suggest a speculative scenario consistent with the Boolean function of Figure 3C: The absence of *NCF4* (perhaps as a result of mutation), if accompanied by other unknown factors, permits activation of the NADPH oxidase, which could be aberrant, i.e., not necessarily responding to the presence of invasive microorganisms. If this happens, then the resulting oxidative burst, evidenced by *PGM1* down-regulation, is damaging to the cell and is normally accompanied by triggering apoptotic mechanisms, which, however, are inhibited by the activated *HGF* resulting from the presence of hepsin. The damaged surviving cell may then become cancerous as a result of additional mutations. This interpretation may also shed light on the problem of the “hepsin paradox” [27]: Although hepsin is overexpressed in the vast majority of prostate cancers and is thought to promote tumor progression, it is unexpectedly underexpressed in metastatic lesions. It could be that the additional mutations in the damaged cells have already inactivated the apoptotic mechanisms, at which stage the expression of hepsin is not needed anymore for the “protection” of the cell, and cancer becomes more invasive. Furthermore, as noted above, the same conditional entropy (0.1159) with the same Boolean function results if we replace gene *NCF4* with gene *KRT6E (keratin 6E)*. It is known that mutations in keratin genes can prime cells to oxidative injury [28]. In that case, *KRG6E* is absent due to its mutation, and the resulting oxidative injury is not stemming from NADPH oxidation, but is still manifested by the absence of *PGM1,* and the apoptotic mechanisms are still inhibited by the presence of hepsin.

There are many more gene modules that are revealed by EMBP analysis in addition to those indicated in Figure 3, and their complete presentation and interpretation is beyond the scope of this paper. We note, however, that the “theme” of oxidative stress combined by inhibition of apoptosis is encountered in several of them. Therefore, the credibility of this speculative interpretation is strengthened, because it can be consistently made multiple times with respect to other gene modules. Confirming the plausibility of this hypothesis, independent studies have also linked oxidative stress to prostate cancer [29]. Here we present one more such gene module that we found, which is particularly relevant in that respect.

This module resulted from a threshold choice of 15. As seen in Figure 2A, the conditional entropy of the optimum three-gene module (shown outside the dotted box) is relatively high, exceeding 0.3. Therefore, we found the lowest-entropy four-gene module. The result is shown in Figure 4, and is the one that we had also used to generate Table 1 with corresponding minimum entropy of 0.088. Gene a is *COL4A6 (collagen type IV alpha 6),* gene b is *CYP1B1 (cytochrome P450, family 1, subfamily B, polypeptide 1),* gene c is *SERPINB5 (maspin, or serpin peptidase inhibitor, clade B, member 5),* and gene d is *GSTP1 (glutathione S-transferase pi)*. The most parsimonious Boolean function containing the entries of the Karnaugh map predominantly associated with cancer is (b+d′)a′c′.

**Figure 4:**
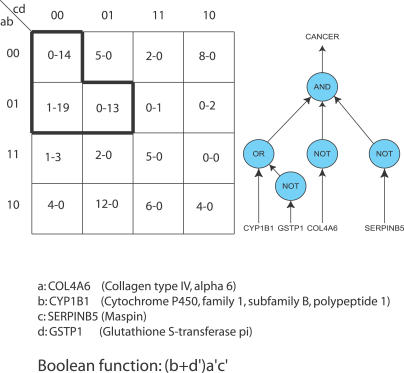
Karnaugh Map and Tree Representation of the Most Parsimonious Boolean Function for the Minimum Entropy Four-Gene Module at Threshold = 15 Cancerous tissues are associated with the absence of both *COL4A6* and *SERPINB5* accompanied by either the presence of *CYP1B1* or absence of *GSTP1*.

The meaning of this function is the following: Cancerous tissues are associated with the absence of both *COL4A6* and *SERPINB5* accompanied by either the presence of *CYP1B1* or absence of *GSTP1*. Figure 4 also shows the Boolean function represented by a tree structure, which can be useful for providing insight into the flow of information in pathways when the number of genes in a module is high.

The common theme in this cluster is easily detected by the simultaneous presence of two genes, which have the following products: On the one hand, CYP1B1 is an oxidative enzyme induced by, and metabolizing, various substances, several of which are toxins, and on the other hand GSTP1 is another enzyme whose role is to detoxify by catalyzing conjugation of glutathione. Products of oxidative metabolism are “natural” substrates for the glutathione transferases [30]. Therefore, consistent with the observation that CYP1B1 and GSTP1 are coordinated in sequential reactions [31], it is reasonable to assume that, in normal prostate tissues, part of the role of GSTP1 is to respond to oxidative stress by detoxifying procarcinogens that have been activated by oxidative enzymes such as CYP1B1.

*CYP1B1* has been found overexpressed and regulated by promoter hypomethylation in prostate cancer [32] and several cancer therapeutic options associated with *CYP1B1* are under consideration [33]. On the other hand, *GSTP1* has been found underexpressed in prostate cancer, often as a result of hypermethylation of its regulatory sequences [34,35]. Interestingly, there are three known gene variants of *GSTP1,* and the gene variant (GSTP1*C) that we include in the Boolean function is the one, among the three, that was shown to contain functional retinoic acid response elements (RAREs) in its introns, confirmed by the observation that retinoic acid treatment significantly increased GSTP1*C gene expression in glioblastoma cells, a fact that may contribute to a better understanding of the molecular regulation of the *GSTP1* gene in human cells [36] and provide possibilities for therapeutic intervention. We note that we have identified gene *RBP1 (retinol binding protein 1)* as a member of several of the gene modules that we found (one of which also appears in Figure 3), and it was recently hypothesized that the protective role of *RBP1* is due to inhibition of the PI3K/AKT survival pathway through a retinoic acid receptor–dependent mechanism [37].

A third molecule in the module of Figure 4 is *SERPINB5,* or *maspin,* a serine protease inhibitor. Its product has been identified as a tumor suppressor, originally in breast cancer [38]. The absence of its expression has also been linked to promoter methylation [39]. Given the identified theme of the gene module, we would like to see in what ways maspin is related to oxidative stress. Strikingly, precisely this role has recently been proposed for maspin in prostate cancer tissues [40]: that maspin may inhibit oxidative stress–induced generation of reactive oxygen species (such as free radicals) by interacting with glutathione S-transferase, thus preventing adverse effects on tumor genetics. This hypothesis is consistent with the entries of Figure 4, indicating that a combination of *GSTP1* and *SERPINB5,* and not *GSTP1* alone, as noted earlier, is needed to counteract the expression of *CYP1B1*. Indeed, although all 13 tissues associated with state 0101 are cancerous (*N*_0_ = 0 and *N*_1_ = 13), all five tissues associated with state 1111, in which maspin is expressed in addition to *GSTP1,* are healthy (*N*_0_ = 5 and *N*_1_ = 0).

More specifically, maspin was found to interact with three molecules: The two molecules in addition to glutathione S-transferase were two “stress proteins” (heat shock proteins HSP70 and HSP90), suggesting that intracellular maspin may be primarily involved in cellular response to stress stimuli [40] by inducing apoptosis [41]. Furthermore, heat shock proteins are known to be induced by oxidative stress and they may play oncogenic roles by “protecting” cells from apoptosis. For example, it has been found that reactive oxygen species play important roles in the activation of HSF1 (heat shock factor 1, the primary transcription factor responsible for the transcriptional heat stress response in mammalian cells) and in the accumulation of mRNA of the previously mentioned genes *HSP70* and *HSP90* in the ischemic-reperfused heart [42].

From the observations so far, it follows that GSTP1 and maspin may work synergistically to counteract oxidative stress, perhaps by maspin inducing apoptosis whenever GSTP1-induced detoxification is not successful. Interestingly, maspin, a serine protease inhibitor, and hepsin, a serine protease that we previously speculated to play an apoptosis-inhibitive role, were found to be inversely expressed in prostate cancer [43]. Even without the presence of *CYP1B1,* the simultaneous absence of both GSTP1 and maspin appears to be sufficient to cause cancer, as indicated by the entries at states 0000 and 0100 in Figure 4.

The only remaining gene in that module is *COL4A6,* which encodes one of the six subunits of type IV collagen, the major structural component of basement membranes. The association that we found, using EMBP analysis, between *COL4A6* and prostate cancer is remarkable, because *COL4A6* expression is missing in nearly all cancerous tissues as evidenced by the Boolean function. This absence of *COL4A6* expression in prostate cancer has also been observed before [44].

There are two identified splice variants of *COL4A6:* splice variant A and splice variant B, each of which uses a distinct promoter [45]. The specific gene in our microarray data is splice variant B. It was recently found [46] that splice variant B is regulated by HSF1 (heat shock factor 1, mentioned earlier) [42]. Specifically, HSF1 binding was detected at the promoter of splice variant B [46], suggesting that *COL4A6* plays a novel role in the heat shock response. Therefore, the possibility exists that one promoter confers tissue specificity for *COL4A6'*s role in the basement membrane and the other promoter regulates the gene for its potentially novel role [46]. This novel role is unclear, but collagen IV has already been found to regulate pathways related to oxidative stress through ERK activation [47,48].

Together with the fact, noted earlier, that maspin interacts with *HSP70* and *HSP90,* these observations further strengthen the case that the gene module is related to oxidative stress including heat shock–related stress. Interestingly, *HSP70* has been found [49] overexpressed in some prostate cancers and in the plasma levels in prostate cancer patients [50].

Therefore, a plausible speculative scenario for this module is the following: *CYP1B1* expression is induced by some toxins and, at least in some *CYP1B1* polymorphisms [51], they are activated and contribute to a type of cancer-causing oxidative stress. This oncogenic effect can be countered by the expression of *GSTP1* and *maspin,* resulting in either detoxification or apoptosis. In particular, absence of *maspin* may inhibit the apoptotic mechanisms normally activated in these cases. The role of the prominent observed down-regulation of *COL4A6* in the cancerous tissues is unclear, but it may be related to the activation of the heat shock response as a result of the oxidative stress, and it may indicate that collagen plays an important and yet unrecognized role in prostate cancer.

## Discussion

The most notable feature of the EMBP method is that it is systems-based, in the sense that it considers the synergistic contributions of sets of genes, rather than individual genes, or sets of co-regulated genes as in microarray clustering techniques. As a result, the optimal gene module of size *n* − 1 may not be a subset of the optimal gene module of size *n,* because the *n* members of the latter module may interact synergistically toward predicting disease in a manner that cannot be achieved if any one of the *n* members is removed. EMBP analysis actually discovers such gene modules mechanistically, and can therefore complement other gene-set–based techniques, such as “Gene Set Enrichment Analysis” [4], in which the gene sets are defined based on prior knowledge, such as published information about biochemical pathways.

Discrimination between healthy and diseased tissues, or between different types of diseases, is not the main purpose of EMBP analysis, although it can be used for this task. If we wanted to focus on classification, then we would use many genes, connect their expression levels in sophisticated ways, rather than through one extremely simple Boolean function, and use their continuous expression levels instead of binarizing them, so that we can extract additional information. Nevertheless, we still validated our results for classification accuracy using the binarized data from sets of three genes connected by simple Boolean functions, applied on totally new microarray data, independently generated in a different lab. The resulting high classification accuracy of typically more than 90% (Table 4), confirms that the extracted logic function connecting these genes is valid in the testing set as well, and therefore it is likely to be a universal property in prostate cancer tissues. In particular, the “notable facts,” mentioned earlier, resulting from our Boolean functions, indicate a precise functional relationship among the genes leading to disease, which is valid on both the training and the testing datasets.

We tested the effect of sample size on the results by performing EMBP analysis on several randomly selected subsets of the training data using equal numbers (10, 20, 30, and 40) of samples from normal and cancerous tissues. The minimum entropy results are shown in Figure 5. We observe that the average minimum entropy values for *n* = 1, 2, 3, and 4 increase with the number of samples. Furthermore, it is also seen that for lower number of samples, the entropy reaches zero for lower values of *n* when compared with higher sample datasets. However, the entropy values appear to increase at a low rate as *n* becomes close to 50. Thus, we expect that for prostate cancer analysis, having more than a 100 samples from each category would not add additional information.

**Figure 5:**
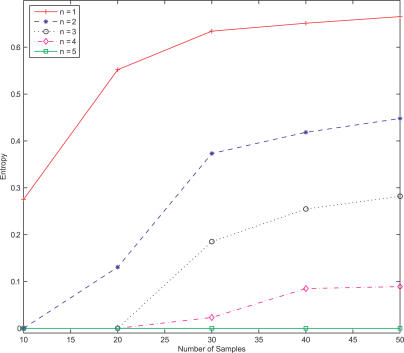
Minimum Entropy Values Averaged over Four Trials across Different Sample Sizes for Threshold = 15 The minimum entropy values increase with sample size for any given *n,* but they tend to saturate for large numbers of samples.

As mentioned earlier, the genes in the modules from EMBP analysis are not co-regulated, but they are co-operative. This is in sharp contrast to the gene modules resulting from clustering or bi-clustering approaches [52–58], the very nature of which is to cluster genes with similar expression patterns. Several of these clustering approaches use entropy extremization and other information theoretic measures, but in totally different contexts compared with our purposes.

For example, in reference [56], entropy minimization is used to reduce the amount of “disorder” within each cluster so that data points within a cluster are similar to each other. In reference [57], the mutual information between different clusters is minimized so that expression profiles falling into different clusters are maximally different from each other. In reference [58], pairwise mutual information is maximized to cluster genes together so that the genes within each cluster have maximum relevance with each other. In all such clustering approaches, the principal strategy is to cluster together genes that have similar expression levels given a particular phenotype. In contrast, the genes within an EMBP module are chosen such that we minimize the uncertainty of assigning correct phenotype labels from the joint expression values of the genes in that module. Thus the genes within the EMBP gene set can have very different expression patterns given the phenotype.

We believe that EMBP analysis provides an opportunity for fruitful cross-disciplinary collaboration in which biologists use the “clues” resulting from the computational results to infer potential pathways, which they can validate with genetic experiments, as well as suggest further computational experiments. For example, if we wish to identify which genes play synergistic roles with another particular gene in terms of causing disease, we could “freeze” the presence of that gene and identify the other genes in a module minimizing the entropy. Furthermore, our systems-based approach can immediately suggest novel potential therapeutic methods that would not be possible with traditional individual-gene approaches, for example, targeting simultaneously two genes that appear in the same Boolean function by combining two already existing drugs targeting each of these genes.

We hope that EMBP analysis will prove to be a significant new tool for medical research working synergistically with the future efforts of diseased tissue genome sequencing. To illustrate with an example, if a Boolean function a′b′c′ is found when analyzing expression data of a particular cancer, this would suggest the possibility that the three genes “gene a,” “gene b,” and “gene c” may cause cancer when all are inactivated, perhaps due to their mutation or to hypermethylation of their promoter, as we previously discussed regarding *KRT6E* and *GSTP1*. In turn, this observation may provide motivation to sequence these genes in cancerous tissues. For EMBP analysis to be significantly effective, in addition to diseased tissues, hundreds of healthy tissues must also be profiled for each tissue type. Inclusion of data from healthy tissues has so far not been emphasized, mainly because microarray data were thought to be more useful for classifying among disease subtypes, rather than detecting disease from non-disease, which is often achievable by normal biopsies. Furthermore, it is important to use large and high-quality input datasets obtained under standardized conditions.

## Materials and Methods

### Entropy minimization.

We used a combination of two heuristic optimization techniques to search for minimum entropy gene sets, allowing sufficient time for each of them to converge. The first technique is the following: Starting from a randomly chosen gene set of size *n,* at each step of the iteration, we modify the “current” gene set by replacing one of its genes, chosen at random, with a new gene, also chosen at random from the entire gene set *M*. If the conditional entropy of the new gene set is lower than that of the current gene set, then the new gene set replaces the current gene set. The process terminates when the conditional entropy is 0, or when the current gene set remains unmodified for a particular large number of steps. To avoid selecting a local minimum, we repeat the same iterative algorithm several times, starting from different initial conditions of the same size, and select the gene set that yields the overall lowest conditional entropy. We then increase the size of the gene set to *n* + 1 and repeat the whole process, making sure that one of the chosen initial conditions contains the previously found gene set. This technique typically converges to some choice of near-optimum results.

To reduce the chance that the found solution corresponds to a local, rather than global minimum, we also used simulated annealing [59] to search in the space of all subsets of size *n*. In an “annealing” process, a melt, initially disordered and at high “temperature” *T,* is slowly cooled. As cooling proceeds, the system becomes more ordered and approaches a “frozen” state at *T* = 0. In our case, we started from a randomly chosen gene set of size *n* and replaced a randomly chosen gene in the set by another randomly chosen gene from the entire set of *M* genes. If the conditional entropy of the new gene set is found to be lower than that of the current gene set, we replace the current set with the new set. If, however, the conditional entropy of the new gene set is higher than that of the current gene set, we allow replacement of the current gene set with a probability *p_—_accept* that is proportional to the temperature *T_k_* at the time and inversely proportional to the amount by which the conditional entropy of the new gene set is higher than that of the current gene set. Thus, the value of *p_—_accept* at any given temperature *T_k_* is given by:

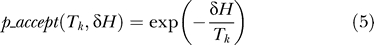
where *δH* is the increase in conditional entropy due to the random change in the gene set.

The parameters of the simulated annealing algorithm that we used were:

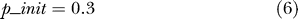
which is the initialization value of *p_—_accept*),

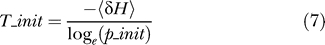
where 〈*δH*〉 is the average increase in conditional entropy due to a random change in a gene set,

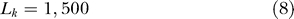
which is the total number of transitions at the *k^th^* temperature,

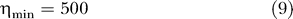
which is the minimum number of acceptances at the *k^th^* temperature, and

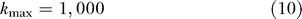
which is the total number of temperature values to be tried over the course of the algorithm.

The “cooling scheme” defines the rate at which the temperature falls over the length of the algorithm. We adopted the exponential cooling scheme defined as *T_k+1_ = αT_k_,* where *α* = 0.98. The simulated annealing algorithm allows for significant increases in the conditional entropy at higher temperatures, searching coarsely in the space of subsets of genes. As the temperature falls, the probability of accepting even small increases in conditional entropy is reduced, thus searching only in the local neighborhood of the conditional entropy value.

An average run of the simulated annealing algorithm for estimating the minimum conditional entropy gene-set for *n* = 3, on a Pentium III processor running at 3 GHz is around 30 min.

## Supporting Information

### Accession Numbers

The National Center for Biotechnology Information (NCBI) (http://www.ncbi.nlm.nih.gov) accession numbers for the genes discussed in the paper are as follows: *COL4A6* (D21337), *COL4A6* splice variant A (NM_001847), *COL4A6* splice variant B (NM_033641), *CYP1B1* (U03688), *DF* (M84526), *ENTPD1* (AJ133133), *FNBP1* (AB011126), *GSTP1* (U12472), *HIST1H1E* (M60748), *HLA-DQB1* (M81141), *HPN* (X07732), *KRT6E* (L42611), *MCM3AP* (AB011144), *NCF4* (AL008637), *NELL2* (D83018), *PGM1* (M83088), *RBP1* (M11433), *SERPINB5* (U04313), *SPINK2* (X57655), and *TMSL8* (D82345).

## Abbreviations

EMBP: Entropy Minimization and Boolean Parsimony
